# Therapeutic DNA Vaccine Encoding Peptide P10 against Experimental Paracoccidioidomycosis

**DOI:** 10.1371/journal.pntd.0001519

**Published:** 2012-02-28

**Authors:** Glauce M. G. Rittner, Julián E. Muñoz, Alexandre F. Marques, Joshua D. Nosanchuk, Carlos P. Taborda, Luiz R. Travassos

**Affiliations:** 1 Institute of Biomedical Sciences, Department of Microbiology, University of São Paulo, São Paulo, São Paulo, Brazil; 2 Departments of Medicine, and Microbiology and Immunology, Albert Einstein College of Medicine, Bronx, New York, United States of America; 3 Medical Mycology Laboratory-IMTSP and LIM53/HCFMUSP, University of São Paulo, São Paulo, São Paulo, Brazil; 4 Cell Biology Division, Department of Microbiology, Immunology and Parasitology, Federal University of São Paulo (UNIFESP), São Paulo, São Paulo, Brazil; George Washington University, United States of America

## Abstract

Paracoccidioidomycosis (PCM), caused by *Paracoccidioides brasiliensis*, is the most prevalent invasive fungal disease in South America. Systemic mycoses are the 10th most common cause of death among infectious diseases in Brazil and PCM is responsible for more than 50% of deaths due to fungal infections. PCM is typically treated with sulfonamides, amphotericin B or azoles, although complete eradication of the fungus may not occur and relapsing disease is frequently reported. A 15-mer peptide from the major diagnostic antigen gp43, named P10, can induce a strong T-CD4+ helper-1 immune response in mice. The TEPITOPE algorithm and experimental data have confirmed that most HLA-DR molecules can present P10, which suggests that P10 is a candidate antigen for a PCM vaccine. In the current work, the therapeutic efficacy of plasmid immunization with P10 and/or IL-12 inserts was tested in murine models of PCM. When given prior to or after infection with *P. brasiliensis* virulent Pb 18 isolate, plasmid-vaccination with P10 and/or IL-12 inserts successfully reduced the fungal burden in lungs of infected mice. In fact, intramuscular administration of a combination of plasmids expressing P10 and IL-12 given weekly for one month, followed by single injections every month for 3 months restored normal lung architecture and eradicated the fungus in mice that were infected one month prior to treatment. The data indicate that immunization with these plasmids is a powerful procedure for prevention and treatment of experimental PCM, with the perspective of being also effective in human patients.

## Author Summary

Paracoccidioidomycosis (PCM) is the predominant systemic mycosis in Latin America causing half of the total deaths among systemic fungal infectious diseases in Brazil. Chemotherapy is the standard treatment, but the long time required, severe cases of immunosuppression and frequent relapses indicate that additional methods should be introduced such as immunotherapy combined with antifungal drugs. Previously, the protective activity of P10, a peptide derived from the major diagnostic antigen gp43, was demonstrated, alone or combined with chemotherapy. P10 elicited a vigorous IFN-γ mediated Th-1 immune response. Presently, the reduction of fungal load, and even sterilization, was attempted using a specific DNA vaccine encoding P10. Plasmid pcDNA3 expression vector with P10 insert was tested as a vaccine in intratracheally infected BALB/c and B10.A mice. Our results showed that vaccination with pP10 induced a significant reduction of the fungal burden in the lung. Co-vaccination of pP10 with a plasmid encoding mouse IL-12 proved to be even more effective in the elimination of the fungus with virtual sterilization in a long term infection and treatment assay system. The data suggest that immunization with these plasmids, without the need of an adjuvant, could be used in the prevention and treatment of PCM in human patients.

## Introduction

*Paracoccidioides brasiliensis* is a thermally dimorphic fungus that causes a systemic granulomatous disease known as paracoccidioidomycosis (PCM). PCM is widespread in Latin America, mainly affecting rural workers, and its incidence has increased in recently deforested areas associated with soil churning [1]. Acquisition of *P. brasiliensis* may arise from inhalation of aerosolized conidia.

Recently we reviewed the death rates by systemic mycoses in Brazil [2]. PCM was the principal cause of death identified for 3,583 patients in the 1996–2006 decade and representing 51.2% of total deaths due to systemic mycoses. It ranks as the 10^th^ most common cause of death among infectious diseases in Brazil [2].

There are distinct forms of PCM. The acute and sub-acute forms affect both genders with primary involvement of the reticuloendothelial/lymphatic system. The chronic form affects mainly adult males and predominantly causes pulmonary and/or mucocutaneous disease [3]. Antifungal chemotherapy is required for treatment, though treatment may not assure complete eradication of the fungus, with frequent relapses. Treatment with itraconazole usually takes 6–9 months in the low and 12–18 months in the moderately severe cases. Frequently, a combination of trimethoprim and sulfamethoxazole (TMP/SMZ) is used, held for 12 months in the low severity forms and 18–24 months in the moderately severe forms. Patients with severe PCM forms require endovenous treatment with anfothericin B or the TMP/SMZ association for long periods, monitored by clinical, radiological and serological tests [4].

The 43 kDa glycoprotein was characterized as the major diagnostic antigen of *P. brasiliensis* [5], [6], [7]. Immunization with gp43 elicited delayed hypersensitivity reactions in guinea pigs [8] and humans [9], implying the presence of T-CD4+ reacting epitopes. Based on the sequence of gp43 [6], which encodes a polypeptide of 416 amino acids with a single high mannose *N*-glycosylated chain [10], the T-cell epitope was mapped to a 15-mer peptide called P10 [11]. The hexapeptide HTLAIR comprises the essential core of P10 that induces proliferation of lymph node cells from mice sensitized to gp43 or infected with *P. brasiliensis* [11]. Type 1-T helper lymphocytes producing IL-2 and IFN-γ are induced by P10 [11], [12], [13]. Intratracheally infected mice previously immunized with P10 in the presence of complete Freund's adjuvant (CFA) had >200-fold reduction of lung *P. brasiliensis* colony-forming units (CFUs). In many cases the immunization rendered preserved lung architecture with few or no yeasts, whereas the infected, unimmunized mice displayed dense pulmonary inflammation characterized by epithelioid granulomas with numerous yeast cells [11], [12].

The immunoprotection by P10 depends on the IFN-γ-producing Th-1 response since mice deficient in IFN-γ, IFN-γ-R or IRF-1, but not IFN-α-R/IFN-β-R, were not protected by P10 immunization [12] The essential role of IFN-γ in organizing granulomas that contain *P. brasiliensis* yeasts has also been recognized by other investigators [13], [14], [15].

Several experimental avenues have been pursued to validate P10 as a vaccine candidate. These studies have included: a) the presentation of P10 by MHC molecules from different murine haplotypes [11]; b) its conservation in nature, confirmed by examining gp43 molecules from different isolates [16]; c) its immunogenicity and effective immunoprotection in formulations that do not require complete Freund's adjuvant [17]; d) its presentation by most human HLA-DR molecules as well as that of neighbor peptides to P10, based on the sequence of gp43 [18]; and e) the effectiveness of P10 as an adjuvant to chemotherapy in normal [19] and anergic [20] mice challenged intratracheally with virulent *P. brasiliensis*.

The immunoprotective properties of P10 emulsified in Freund's adjuvant have been well documented in an established murine model of PCM [11]. Since CFA is not allowed in human vaccines and a tetramer of truncated P10 although immunogenic, involves laborious chemical methods [17], we have explored alternative approaches for P10 delivery. In the present work we have investigated the effectiveness of plasmid immunization with P10 and/or IL-12 inserts given prior to or after challenge with a virulent Pb18 isolate of *P. brasiliensis* using a murine pulmonary PCM disease model. Our results demonstrate that plasmid immunization with P10 with or without IL-12 inserts is highly therapeutic in mice intratracheally infected with this fungus. Most importantly, immunization was effective either prior to, or after infection suggesting that these plasmids are candidates for use in human PCM.

## Materials and Methods

### Plasmid constructions

Yeast cells of *P. brasiliensis* isolate 18 (Pb18) were grown in Sabouraud Dextrose Broth (BD, MD, USA) at 37°C for 7 days. Cells were washed and frozen in liquid nitrogen then disrupted by grinding on a mortar. Total RNA was isolated with Trizol according to manufacturer's instruction (Invitrogen, CA, USA). Complementary DNA was synthesized from 1 µg of total RNA in the presence of oligo(dT)_18_ (Fermentas, MD, USA) and Revertaid M-MuLV(Fermentas, MD, USA).

The P10 nucleotide sequence was obtained using the sense PCR primer derived from the gp43 [6] 5′ nucleotide sequence: [5′-AAT AAG CTT CAA ACC CTG ATC GCC-3′], and the antisense primer derived from the 3′ end of the gp43 gene: [5′- AAT GAA TTC ATT GGC GTA ACG GAT TGC-3′]. A HINDIII site and an EcoRI site were added to the sense and antisense primers, respectively, for cloning into plasmid pcDNA3 (Invitrogen, CA, USA). PCR reactions (50 µl) were carried out following the protocol provided by Fermentas, using 100 ng of cDNA and 100 ng of each primer. The P10 PCR reaction started with one cycle at 94°C (2 min), followed by 40 cycles at 94°C (30 sec), 55°C (1 min) and 72°C (1 min), and a final 7-min extension at 72°C. PCR products were purified using Wisard SV gel and PCR Clean-UP system (Promega, Brazil) and each PCR product was digested with the appropriate restriction enzyme (Fermentas) and cloned into the pcDNA3 by directional insertion in the HINDIII/EcoRI sites. The resulting plasmid was called pP10.

Plasmid pORF-mIL-12 was acquired from InvivoGen (CA, USA). The confirmation of the insert was done using the primers: sense [5′-CGG GTT TGC CGC CAG AAC ACA-3′] and antisense [5′-GGC CAC CAG CAT GCC CTT GT-3′]. The IL-12 PCR started with one cycle at 94°C (2 min), followed by 40 cycles at 94°C (1 min), 45°C (1 min) and 72°C (2 min), and a final 7-min extension at 72°C.

### Preparation of plasmid DNA

To prepare plasmid DNA for immunization, *Escherichia coli* XL1Blue and DH5α cells were transformed by electroporation using Cellject Duo according to the manufacturer's directions (Hybaid, Middx, UK) with the DNA constructs or the vector plasmid alone and then cultured at 37°C in Luria broth supplemented with ampicillin (50 µg/ml).

The positive clones were confirmed by automatic sequencing carried out following the protocol provided by Applied Biosystems (CA, USA) and analyzed by BioEdit and Blast. The parental vectors, pcDNA3 and pORF were used as negative controls. DNA for immunization was purified using the EndoFree Giga Kit (Qiagen, CA, USA) and was diluted in TE buffer to the final concentration of 1 µg/µl.

### Plasmid gene expression in mammalian cells

For the expression of pORF-mIL-12 in mammalian cells, a transient-transfection assay was performed using Lipofectin (Invitrogen) and 1 or 2 µg plasmid transfected into HeLa cells (2×10^5^ cells/well). The cells were grown in RPMI medium supplemented with 10% fetal calf serum (FCS) (Cultilab, SP, Brazil). After 24 h incubation, the cells were harvested, and total RNA was isolated with Trizol for reverse transcription (RT)-PCR. IL-12 PCR was used as described above. IL-12p70 was detected (80 ng/ml) by ELISA, in the supernatant of transfected HeLa cells.

### P10 expression from vector pcDNA3, followed by IFN-γ production

DNA immunization was performed by injecting groups of 5 six-week-old male BALB/c mice intramuscularly in both quadriceps with three doses of 100 µg of plasmid encoding P10 (pP10), 50 µg of either pP10 and pcDNA3 vector alone, or 50 µg of the pcDNA3 vector alone, each in 50 µl of TE buffer. A total of three immunizations were given at weekly intervals in alternating sites on the left and right hind legs. The mice were euthanized one week after the last immunization, their spleens were isolated and single-cell suspensions were prepared by gentle homogenization in RPMI medium supplemented with 1% FCS. Cells were suspended and treated with isotonic ammonium chloride to lyse erythrocytes. The splenocytes were washed by centrifugation, suspended in RPMI containing 10% FCS, and dispensed into wells on a microtitering plate (5×10^5^ mononuclear cells per well). The cultures were stimulated with 20 µg/ml of synthetic P10. After 24 and 48-h incubation at 37°C with 5% CO_2_, supernatants were collected and IFN-γ was assayed by a sandwich enzyme-linked immunoassay (ELISA) (BD Pharmingen, CA, USA). Splenocytes from animals immunized with pP10 and stimulated with synthetic P10 produced 10 and 15 ng/ml IFN-γ after 24 and 48 h, respectively. When 50 µg of pcDNA3 was used for immunization, 9 and 11 ng/ml IFN-γ was released by splenocytes at the two examined times.

### Ethics statement

This study was carried out as recommended by the Brazilian college of animal experimentation (COBEA). The protocol has been approved by the Ethical Committee on Animal Experimentation of University of São Paulo (Permit number: 039).

### Animals

BALB/c and B10.A mice were bred at the Institute of Biomedical Science of University of São Paulo, Department of Immunology animal facility under specific-pathogen-free conditions.

### Fungal strain

Yeast cells of the virulent isolate Pb 18 of *P. brasiliensis* were maintained by weekly subculturing on Sabouraud Dextrose Agar and incubation at 37°C. Before experimental infection, 7–10 day-old cells were inoculated into Sabouraud Dextrose Broth and incubated at 37°C for 5–7days with rotary shaking. Fungal cells were washed three times in phosphate-buffered saline pH 7.2 (PBS) and counted in a haemocytometer. The viability of fungal cells in the inoculum was determined by staining with Janus B (Merck, Darmstadt, Germany) and was greater than 90%.

### Intratracheal infection of BALB/c and B10.A mice

BALB/c and B10.A mice (6- to 8-week-old males) were inoculated intratracheally (IT) with 50 µl suspension of 3×10^5^ Pb18 yeast cells in sterile saline (0.85% NaCl). Mice were anesthetized i.p. with 200 µL of a solution containing 80 mg/kg ketamine and 10 mg/kg of xylazine (both from União Química Farmacêutica, Brazil). After approximately 10 min, their necks were extended to expose the trachea at the thyroid level and cell suspensions were injected with a 26-gauge needle. The incisions were sutured with 5-0 silk.

### Immunization of mice with plasmid DNA

**Figure 1:**
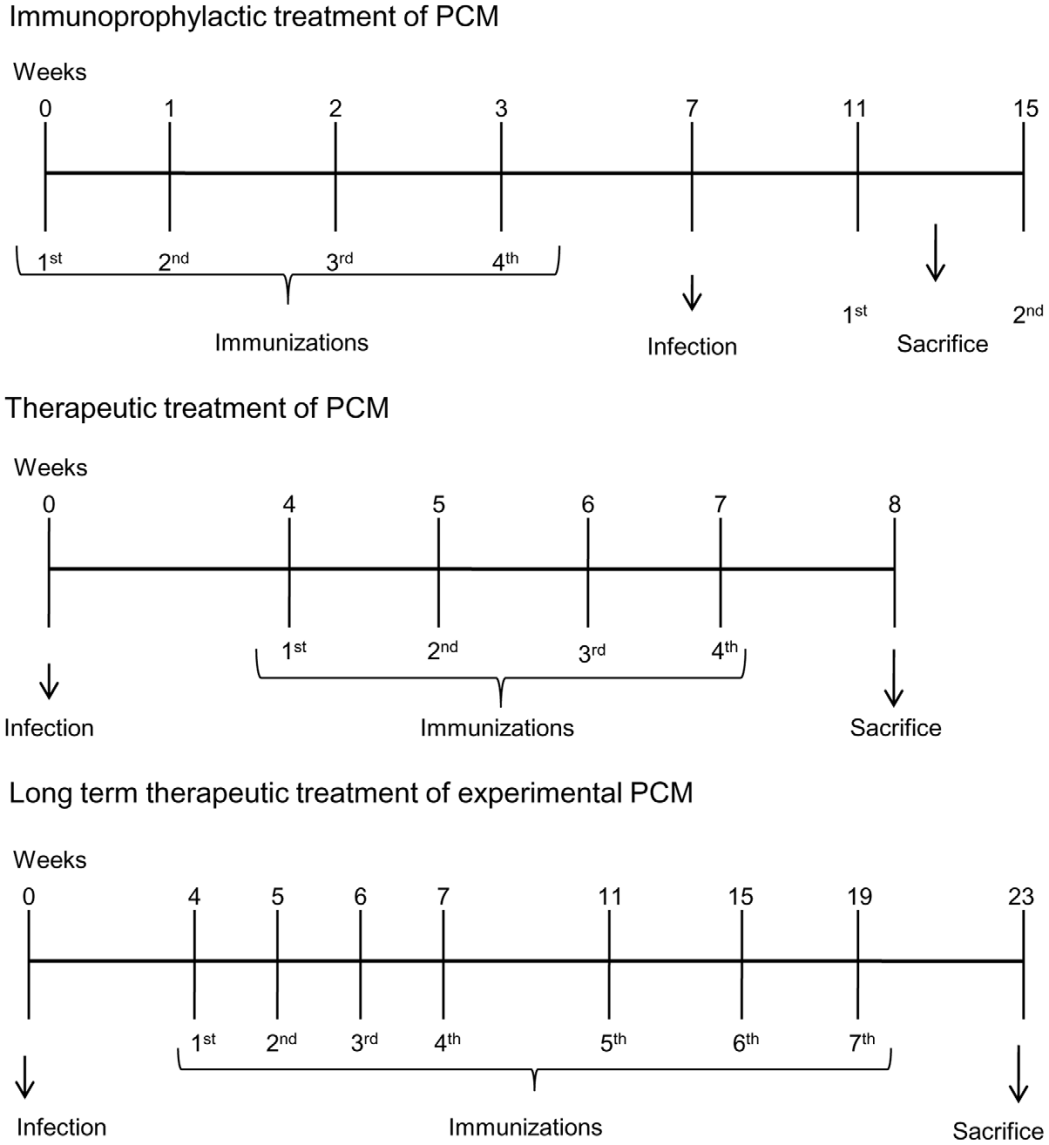
Summary of the treatment protocols used. **First protocol**: BALB/c mice received 4 weekly injections. Animals were infected and sacrificed 30 or 60 days later. **Second protocol**: BALB/c and B10.A mice were infected intratracheally. Mice received 4 weekly vaccine doses and animals were sacrificed 1 week after the last injection. **Third protocol**: B10.A mice were infected i.t. and one month after infection, they were immunized with the DNA vaccine. The animals were sacrificed one month after the last injection, six months after infection.

Three different protocols were used (**Fig. 1**). Injections of 50 µg plasmid were given in the quadriceps muscle. **First protocol**: Groups of 10 BALB/c mice were injected with PBS (control), pcDNA3 (50 µg; control), pORF (50 ug; control), plasmid encoding P10 (pP10, 50 µg), plasmid encoding IL-12 (pIL-12, 50 µg) or with both pP10 and pIL-12 (50 ug each). A total of 4 injections were given weekly on alternating sites, on the left and right hind legs. One week after the last injection, mice were infected intratracheally then sacrificed 30 or 60 days later. **Second protocol**: BALB/c and B10.A mice (10 mice per group) were infected intratracheally. One month after infection, the mice received 4 weekly injections of either PBS (control), pcDNA3 (50 µg; control), pP10 (50 µg), pIL-12 (50 µg) or both pP10 and pPIL-12 (50 ug each). Mice were sacrificed 1 week after the last injection. **Third protocol**: B10.A mice (10 mice per group) were infected intratracheally. One month after infection, they were treated with 4 weekly injections followed by a monthly booster for 3 additional months (total of 7 injections). The injections were with either PBS (control), pcDNA3, pP10, pIL-12 or both pP10 and pPIL-12. The mice were sacrificed one month after the last injection, six months after infection.

### Fungal burden in organs of infected mice

Mice were sacrificed and the lungs, liver and spleen were removed. Weighed tissue sections were homogenized and then washed 3 times with PBS and suspended in 1 ml PBS. Suspensions (100 µl) were inoculated on brain-heart infusion (BHI) agar medium supplemented with 4% FCS and 5% spent culture medium of *P. brasiliensis* (strain-192), streptomycin/penicillin 10 IU/ml (Cultilab) and cycloheximide 500 mg/ml (Sigma, MO, USA). Colonies were counted after 10 days of incubation at 37°C.

### Histopathology

Lung sections from sacrificed mice were fixed in 10% buffered formalin for 24 h and embedded in paraffin. Four-micra sections were stained with haematoxylin-eosin (HE) or silver nitrate (Gomori) and examined microscopically (Optiphot-2; Nikon, Tokyo, Japan).

### Cytokine detection

Sections of excised lungs were homogenized in 2 ml of PBS in the presence of protease inhibitors: benzamidine HCl (4 mM), EDTA disodium salt (1 mM), N-ethylmaleimide (1 mM) and Pepstatin (1.5 mM) (Sigma, St Louis, MO). The supernatants were assayed for IL-4, IL-10, IL-12, and IFN-γ using ELISA kits (BD OpTeia, San Diego, CA). The detection limits of the assays were as follows: 7.8 pg/ml for IL-4, 31.3 pg/ml for IFN-γ and IL-10, 62.5 pg/ml for IL-12, as previously determined by the manufacturer.

### Statistical analysis

Statistical analyses were performed using GraphPad Prism5 software. The results are expressed as means and standard deviations (SD). The nonparametric Kruskall-Wallis honestly significant difference test was used. *p* values are shown in the Figure legends.

## Results

### Colony-forming units (CFU) in mice immunized prior to infection (prophylactic immunization). First protocol

**Figure 2:**
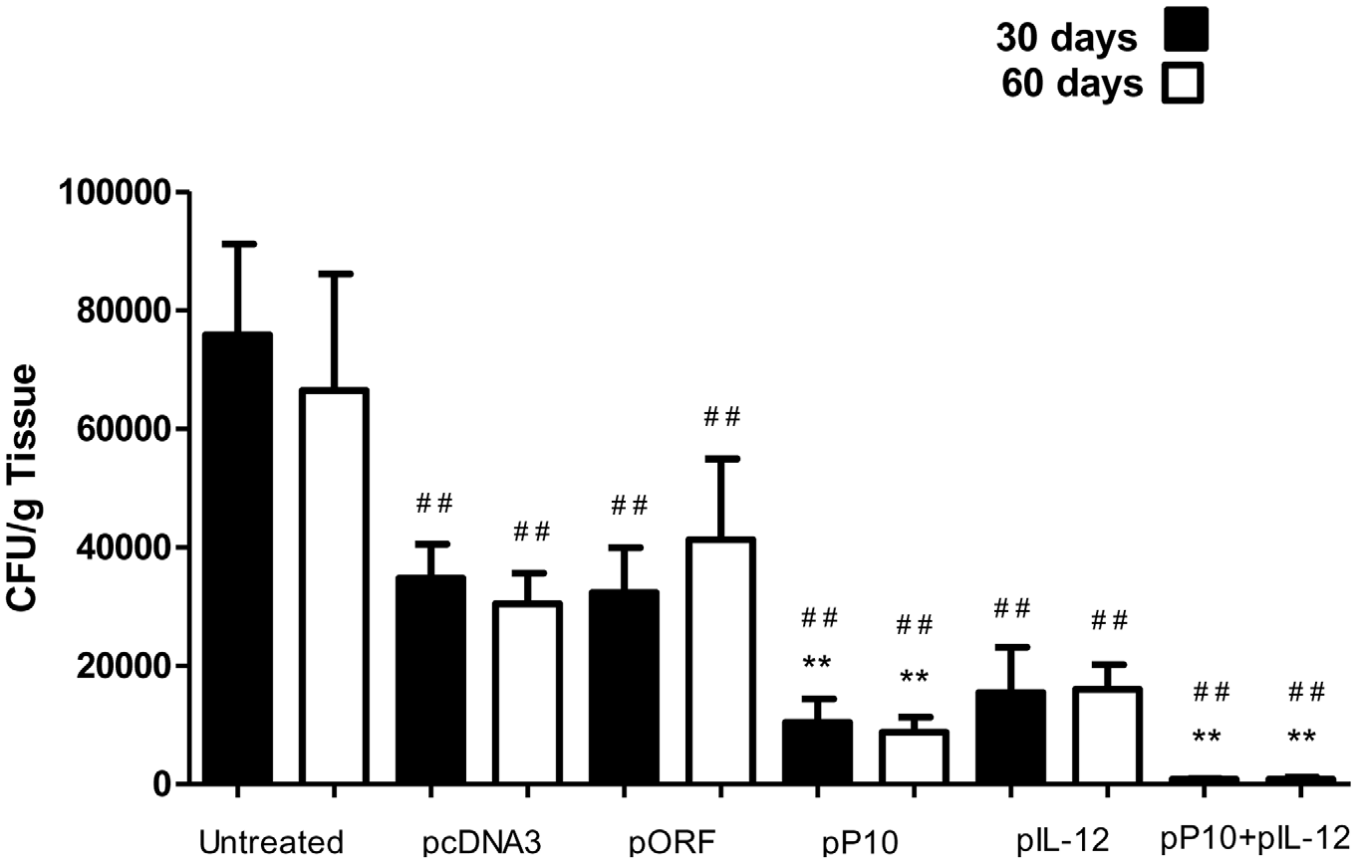
Immunoprophylactic treatment of PCM. Gene immunization was initiated 30 days before fungal challenge. CFUs are from lungs of BALB/c mice infected intratracheally with 3×10^5^ yeast cells and subjected to immunization with vectors containing P10 (pP10) and/or IL-12 (pIL-12) DNA insert. Control mice were inoculated with PBS or with vectors without insert. Mice were sacrificed 30 (▪) and 60 (□) days after infection. Each bar represents the average counts and standard deviations of CFUs in lungs from 10 animals in each group. Experiments were carried out in triplicate with similar results. ** *p*≤0.0001, comparing vector with and without insert; ^##^ *p*≤0.0001, .comparing untreated and other groups.

To explore the effects of the plasmid with the P10 insert (pP10) with or without the murine IL-12 gene insert (pIL-12), BALB/c mice were immunized and then infected intratracheally with virulent *P. brasiliensis* Pb 18. Animals were sacrificed after 30 or 60 days, and the fungal burden in the lungs, spleens and livers was determined. The number of lung CFU per gram of tissue was significantly reduced in animals immunized with pP10 and/or pIL-12 compared to controls at both time intervals (**Fig. 2**). Notably, we observed that the empty plasmids (pcDNA3 and pORF) also induced a significant reduction in CFU relative to mice that received PBS alone, which is presumably a result of dendritic cell activation through Toll-like receptor 9 binding of plasmid unmethylated CpG motifs. Immunostimulation by DNA from *P. brasiliensis* also attributed to CpG motifs showed protective effects in susceptible mice [21], [22]. Nevertheless, the fungal load measured in CFUs in mice receiving pP10 and/or pIL-12 was significantly lower than that in mice treated with control pcDNA3 and pORF. Livers and spleens from all animals had no detectable fungal cells.

### Organ CFUs in mice immunized 1-month after infection (therapeutic immunization). Second protocol

**Figure 3:**
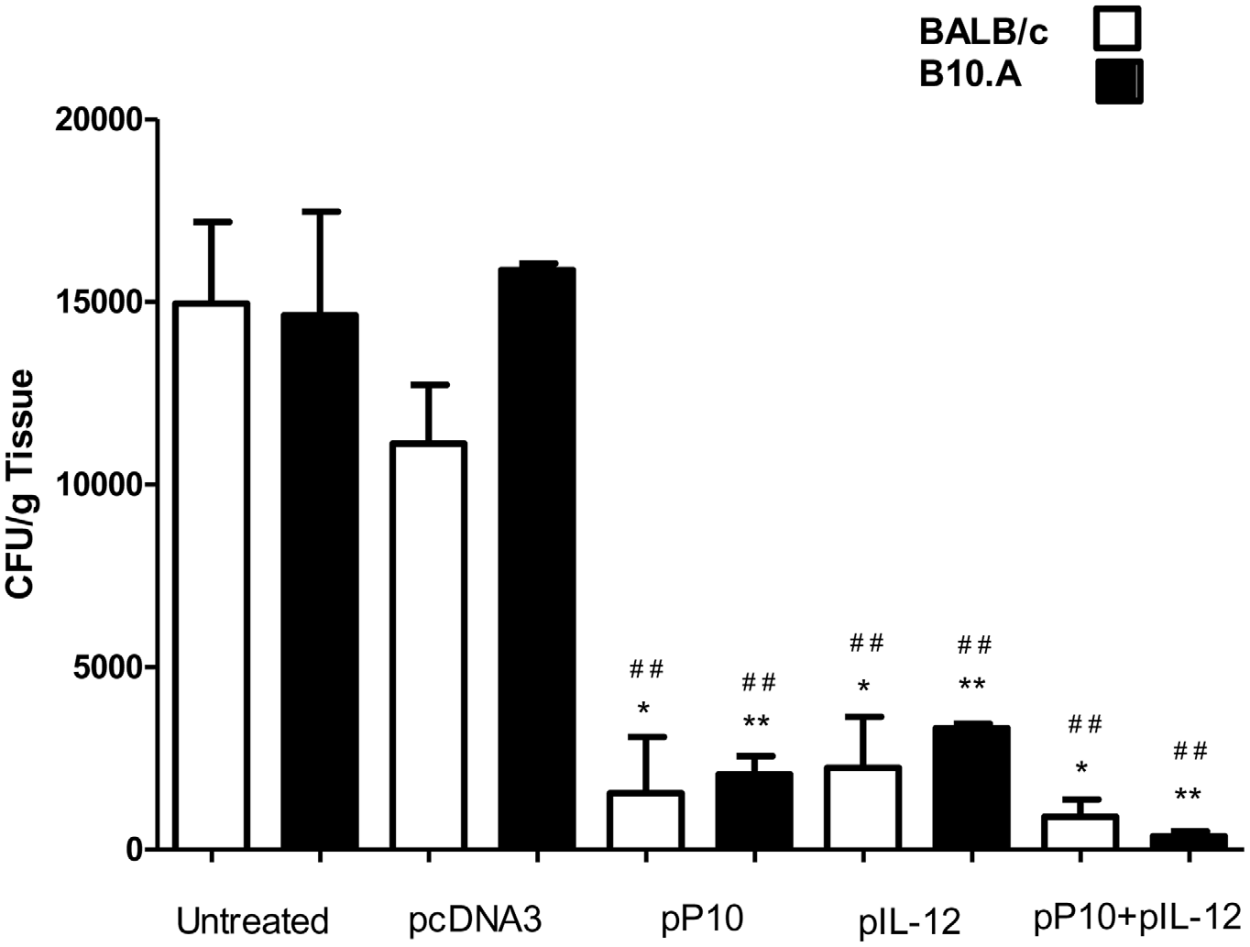
Therapeutic treatment of PCM. Gene immunization started 30 days after infection. CFUs are from lungs of BALB/c (□) and B10.A(▪) mice infected intratracheally with 3×10^5^ yeast cells and subjected to immunization with vectors containing P10 (pP10) or IL-12 (pIL-12) DNA inserts. Control mice were inoculated with PBS or with vectors without insert. Mice were sacrificed 60 days after infection. Each bar represents the average counts and standard deviations of CFU in lungs from 10 animals in each group. Experiments were performed three times and similar results were achieved. * *p*≤0.05, ** *p*≤0.005, comparing vector with and without insert; ^##^ *p*≤0.005, comparing untreated and other groups.

The therapeutic protocol attempts to reproduce the clinical reality of patients presenting to medical attention after developing symptomatic PCM. We studied two mouse strains with different susceptibilities to PCM, BALB/c (susceptible) and B10.A (highly susceptible) [23]. The data showed that immunization with pP10 and/or pIL-12 was therapeutic in mice infected with *P. brasiliensis* for 1 month prior to receiving plasmid immunizations (**Fig. 3**). CFU reductions were significant in infected mice receiving pP10 and/or pIL-12 compared to mice injected with PBS or pcDNA3. In contrast to the first protocol, injection of pcDNA3 after installing PCM was not sufficient to reduce the fungal burden. The most significant reduction in the lung CFUs from B10.A mice was achieved when pP10 and pIL-12 were combined. The CFUs from the livers and spleens were barely detectable in all groups.

### Organ CFUs in a long-term infection model of B10.A mice submitted to gene immunization. Third protocol

**Figure 4:**
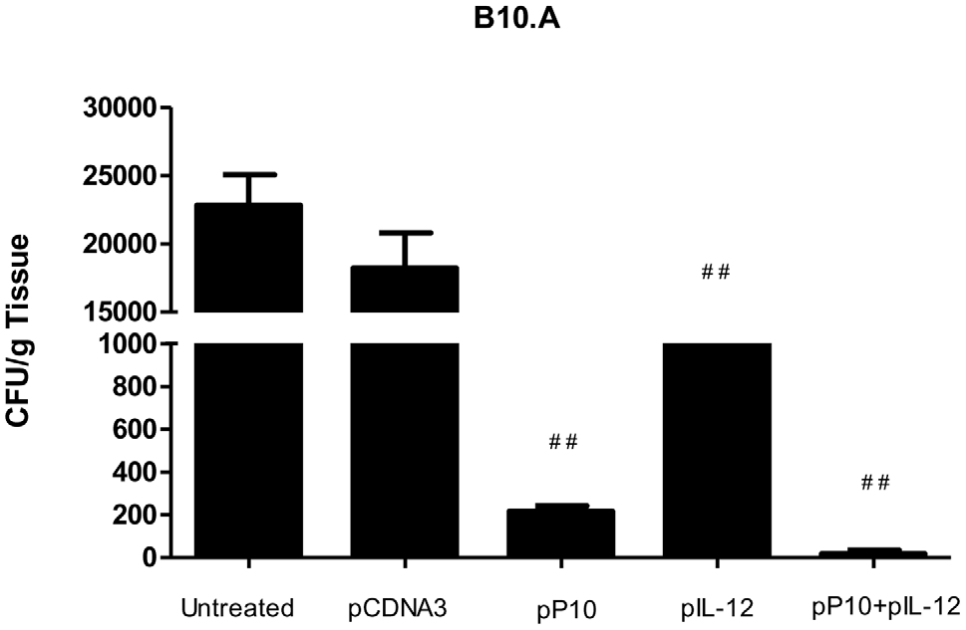
Long term therapeutic treatment of experimental PCM. Gene immunization started 30 days after infection and mice were sacrificed 6 months after infection. CFUs were counted in lungs of B10.A mice infected intratracheally with 3×10^5^ yeast cells and immunized with vectors containing the insert encoding P10 (pP10) or IL-12 (pIL-12). Control mice were inoculated with PBS or with vector without insert. Each bar represents the average counts and standard deviations of CFU in lungs from 5 to 10 animals in each group. Experiments were carried out in triplicate, with similar results. ** *p*≤0.001, comparing vector with and without insert; ^##^ *p*≤0.001, comparing untreated and other groups.

This protocol allowed us to analyze the efficacy of therapeutic plasmid treatment during long-term infection (six months) of the highly susceptible mouse strain, B10.A. Treatment of mice with PCM using pP10 and/or pIL-12 significantly reduced lung CFUs (**Fig. 4**). However, the impact of pIL-12 alone was not as dramatic as either pP10 alone or pP10 with pIL-12. Notably, treatment with the combination of pP10 and pIL-12 virtually eradicated the infection in all organs examined.

### Lung histopathology

**Figure 5:**
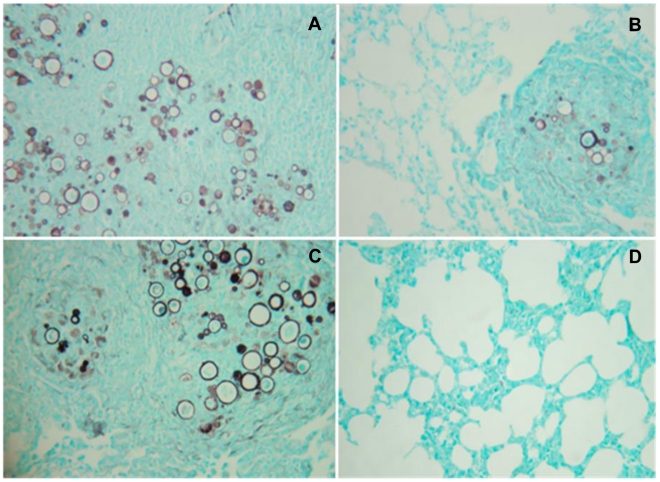
Histopathology of lungs from intratracheally infected B10.A mice. Animals were infected with *P. brasiliensis* for one month, treated with or without vectors carrying P10 or IL-12 DNA inserts according to protocol 3, and sacrificed 6 months after the initial infection. Infected mice treated with (**A**) control pcDNA3, (**B**) pP10, (**C**) pIL-12 DNA, and (**D**) P10 and IL-12 DNA. Gomori staining; original magnification, 40×.

The lungs of control animals in each experimental protocol group showed intense inflammation and large numbers of yeast cells, whereas mice receiving pP10 with or without pIL-12 had significantly reduced inflammation, and lower or undetectable fungal cells. Analysis of the lungs of animals from the third protocol (6 months infection) that received control plasmids revealed dense infiltration of inflammatory cells, mainly of macrophages, lymphocytes and epithelioid cells, and numerous fungal cells (**Fig. 5A**). Around the foci of epithelioid granulomas, giant cells were observed. In contrast, there were large areas of normal lung architecture in pP10-immunized mice and a global reduction in the number of granulomas with few yeast cells (**Fig. 5B**). Treatment with pIL-12 resulted in histological findings that were more similar to controls than to pP10-immunized mice (**Fig. 5C**). Importantly, the lungs of mice treated with the combination of pP10 and pIL-12 were mostly histologically normal and no yeast cells were identified (**Fig. 5D**).

### Cytokine assays

Previous studies with BALB/c mice have established that P10 elicits a protective Th-1 immune response [11]. BALB/c and B10.A mice have different genetic backgrounds that strongly influence their response to infection by *P. brasiliensis*. Their different susceptibility to fungal infection depends in part on their capacity to produce pro-inflammatory cytokines, which are often reduced in B10.A relative to BALB/c. IL-4, IL-10, IL-12 and IFN-γ were measured in the lungs of infected B10.A mice and BALB/c. In mice subjected to the second protocol, BALB/c mice responded to pcDNA3 and pP10 gene immunization with significant increase in IFN-γ in the lung homogenate compared to untreated or pcDNA3 treated animals (data not shown). In contrast, B10.A mice produced significantly less IFN-γ after immunization with pP10 and pcDNA3 in comparison to treatment with pcDNA3 gene alone, which suggests that the increase in IFN-γ in both of these groups relative to untreated mice could be due to dendritic cell activation by plasmid CpGs. The cytokine production in the group of animals submitted to the third protocol, in which B10.A mice treated with pP10 with or without pIL-12 had undetectable yeasts in the lung tissue, is shown in **Table 1**. After 6 months post-infection, cytokine analyses in these mice showed a persistent IFN-γ production regulated by an IL-10-rich immune response, which is compatible with a protective therapeutic effect in B10.A mice.

**Table 1:** Cytokines in lung homogenates of B10.A mice infected i.t. with *P. brasiliensis* Pb 18 yeasts and submitted to gene immunization for 5 months.

Cytokines pg/ml	IL-10	IFN-γ
Untreated	12.18±4.61	4.71±1.75
pcDNA3	10.38±6.53	4.73±2.45
pP10+pcDNA3	18.71±6.10	7.64±2.46
pP10+pIL-12	12.00±2.06	7.31±1.37

Mice were sacrificed 1 month after the last dose.

## Discussion

A vaccine against *P. brasiliensis* using plasmid DNA was first tested in 2000 [24], [25]. BALB/c mice were immunized with a mammalian expression vector carrying the full gene of the gp43 under the control of CMV promoter with Freund's adjuvant resulting in the induction of both B and T cell-mediated immune responses characterized by a mixed Th-1/Th-2 long-lasting cellular immune response, chiefly modulated by IFN-γ. This immunization method was protective when performed in mice prior to challenge with virulent *P. brasiliensis*. When tested for immunoprotection, P10 in Freund's adjuvant was also active in the murine model of PCM, eliciting an IFN-γ-dependent Th-1 immune response [11], [20]. The combined treatment of P10-vaccine in Freund's adjuvant and chemotherapy, either an azole, amphotericin B, or sulfamethoxazole, stimulated a protective Th-1 response, rich in IL-12 and IFN-γ, that was therapeutically beneficial if initiated 2 or 30 days after intratracheal infection [19]. The combined treatment was also effective in anergic animals challenged with the fungus [20].

Presently we used a DNA vaccine encoding P10 with or without a plasmid encoding IL-12 that is administered without adjuvant. We found that the pP10 vaccine, either given prior to or 1 month after intratracheal infection, induced a significant reduction in the fungal burden in the lungs of mice. Co-vaccination with murine pIL-12 significantly enhanced vaccine effectiveness, particularly in a long- term infection model in B10.A mice. The combined DNA vaccine (Protocol 3) achieved virtual sterilization after 6 months with histologically normal lungs and undetectable fungal burden. Full protection was mediated by IFN-γ production and the pro-inflammatory effect of pP10 and pIL-12 was regulated by IL-10 in these susceptible mice.

The mechanism of fungal killing by gene immunization is not solely mediated by cytokines since the empty plasmid pcDNA3 is a strong stimulator of the immune system. However, a significant protection is only achieved with pP10 or pP10+pIL-12 administration. P10 is not protective in IFN-γ-KO mice [12], indicating that this cytokine is essential for fungal killing through macrophage activation. T-CD4+ lymphocytes recognizing P10 and other cells induced by fungal infection are the main producers of IFN-γ. A role for a simultaneous induction of protective antibodies against fungal antigens [26] is also recognized.

IL-12 administration has been previously studied in experimental PCM [27]. Our current results show that IL-12 protected mice against disseminated infection. In the long term infection protocol, pIL-12 alone was only partially effective in the protection of infected mice, but the cytokine facilitated the elimination of *P. brasiliensis* when combined with pP10. This is a very encouraging result and strongly suggests that a pP10-based vaccine associated with pIL-12 could be used as a powerful adjuvant to chemotherapy.

Despite the effectiveness of chemotherapy, fatalities from invasive or systemic fungal diseases are not uncommon. Vaccines against fungal diseases are gaining increasing attention, owing to their capacity to effectively modulate the immune response (reviewed in [28]. The frequent occurrence of clinical relapses and sequellae, such as pulmonary fibrosis, following antifungal chemotherapy suggest that immunoprotective vaccines could also reduce the incidence of these complications [29].

In addition to our work with P10, there have been other notable attempts to develop vaccine strategies for the treatment of PCM. They included a cDNA encoding the antigenic protein rPb27 [30], the recombinant heat shock protein 60 emulsified in adjuvant [31], radioattenuated *P. brasiliensis* yeast cells [32] and *Mycobacterium leprae* DNAhsp65 plasmid in infected BALB/c mice [33]. Braga *et al.* [34] immunized BALB/c mice either with recombinant purified flagellins (FliC) genetically fused with P10 or with the synthetic P10 peptide mixed with purified FliC. A prevailing Th1-type immune response was obtained that reduced *P. brasiliensis* growth and lung damage in infected mice.

From a practical standpoint, the broad use of antifungal vaccines is not realistic when considering the perspective of a large number of infected people relative to the number of individuals who develop the disease. Mycoses caused by dimorphic fungi, such as PCM, coccidioidomycosis, histoplasmosis and blastomycosis, have low incidence as a deep-seated disease. Certain fungal diseases such as cryptococcosis, aspergillosis and candidiasis typically occur in immunocompromised hosts. Hence, targeted prophylactic vaccination may be a more practical approach to control disease. In the case of PCM, immunization of those at highest risk, such as farmers in highly endemic regions would be reasonable. However, we have also demonstrated that the pP10/pIL-12 combination is highly efficacious after PCM has developed. Therefore, immunization could be most useful in combination with standard therapy in PCM patients in order to enhance treatment efficacy, reduce treatment duration and, perhaps, prevent relapses.
